# Computational cognitive modeling and validation of Dp140 induced alteration of working memory in Duchenne Muscular Dystrophy

**DOI:** 10.1038/s41598-020-68381-9

**Published:** 2020-07-20

**Authors:** Rahul Tyagi, Palvi Aggarwal, Manju Mohanty, Varun Dutt, Akshay Anand

**Affiliations:** 1 Neuroscience Research Lab, Department of Neurology, Postgraduate Institute of Medical Education and Research, Chandigarh, India; 2 Indian Institute of Technology, Mandi, Himachal Pradesh India; 3 Department of Neurosurgery, Postgraduate Institute of Medical Education and Research, Chandigarh, India

**Keywords:** Network models, Neuromuscular disease

## Abstract

Duchenne Muscular Dystrophy has emerged as a model to assess cognitive domains. The *DMD* gene variant location and its association with variable degrees of cognitive impairment necessitate identification of a common denominator. Computer architectures provide a framework to delineate the mechanisms involved in the cognitive functioning of the human brain. Copy number variations in the 79 exons of *DMD* gene were screened in 84 DMD subjects by Multiplex Ligation-dependent Probe Amplification (MLPA). DMD subjects were categorized based on the presence or absence of DP140 isoform. The cognitive and neuropsychological assessments were carried out as per inclusion criteria using standard scales. Instance-based learning theory (IBLT) based on the partial matching process was developed to mimic Stroop Color and Word Task (SCWT) performance on Adaptive Control of Thought-Rational (ACT-R) cognitive architecture based on IBLT. Genotype–phenotype correlation was conducted based on the mutation location in *DMD* gene. Assessment of specific cognitive domains in DP140 − ve group corresponded to the involvement of multiple brain lobes including temporal (verbal and visual learning and memory), parietal (visuo-conceptual and visuo-constructive abilities) and frontal (sustained and focused attention, verbal fluency, cognitive control). Working memory axis was found to be the central domain through tasks including RAVLT trial 1, recency effect, digit span backward, working memory index, arithmetic subtests in the Dp140 − ve group. IBLT validated the non-reliance of DMD subjects on recency indicating affected working memory domain. Modeling strategy revealed altered working memory processes in DMD cases with affected Dp140 isoform. DMD brain was observed to rely on primacy than the recency suggesting alterations in working memory capacity. Modeling revealed lowered activation of DMD brain with Dp140 − ve in order to retrieve the instances.

## Introduction

Duchenne Muscular Dystrophy (DMD) is a well characterized X-linked recessive neuromuscular disorder which predominantly affects males in early childhood^1^. The disease appears with progressive proximal muscle weakness followed by non-ambulatory phase leading to death in the twenties, caused by cardio respiratory complications^2,3^. The lesser known cognitive impairment also develops in DMD in one third of cases due to which DMD has emerged as a model to understand processes and functioning of crucial cognitive domains^4^. Reduction in mean full scale intelligence quotient (FSIQ), by approximately one standard deviation, with respect to the population mean, has remained a consistent finding in the cognitive profile in these patients^5^. DMD is manifested by rearrangement events including deletion, duplication and point mutations in *DMD* gene which create a shift in the reading frame that result in non-functional dystrophin protein^6,7^. *DMD* gene locus produces full length and short sized dystrophin products from seven distinct promoters. Full length dystrophin (Dp427; based on their length in kiloDaltons) is expressed in the tissue specific manner from three proximal promoters in the muscle, brain and purkinje cells whereas short dystrophin isoforms (Dp260, Dp140, Dp116 Dp71 and Dp40) are expressed in various organs from distant upstream promoters^8^. Dp140, Dp71 and an alternatively spliced short isoform Dp40 are reported to be expressed in the various brain regions including cerebral cortex, cerebellum, hippocampal dentate gyrus^9–11^. Human DMD brain studies have also reported deficiency of dystrophin in the post synaptic densities (PSD) of brain^9^. Dystrophin’s interaction in the critical regions of central nervous system (CNS) indicates its role in the maintenance of higher order cognitive functioning. Studies report deficits in verbal intelligence quotient^12^ as a part of general intelligence and non-verbal memory, executive functions^13^, attention, visuo-construction ability^14^, problems with narrative, linguistic and reading skills^15^. We have also reported a non-progressive deterioration of neuropsychological domains^16^. Conflicting and variable degree of cognitive output in DMD necessitates understanding of a common cognitive domain for designing rehabilitation strategies^17^.

Recent studies indicate a profound impact of mutation location on the degree of cognitive alterations attributed to the predicted absence of associated dystrophin isoform^18–20^. Whether variation in dystrophin expression pattern, affects cognitive domains in phenotypically normal population, has remained an outstanding question. One study reported the role of single nucleotide *DMD* variants (rs147546024:A > G, an intronic variant; rs1800273:G > A, a missense variant) in cognitively healthy general population^21^. A case study reported manifestation of intellectual disability without usual muscular dystrophy phenotype due to deletion of three base pairs (c.9711_9713del) in the distal region of *DMD* gene^22^, providing substantial evidence regarding *DMD* distal region’s association with human cognition.

One way to study human cognition is an emerging discipline which uses algorithmic specificity to design a model similar to human mind and enables analysis of multiple neuropsychological scores. Algorithms work on the hypothesis that provide an artificial cognitive architecture mimicking human mind in the computer systems. Instance-Based Learning Theory (IBLT) was developed to explain human decision-making behaviour based on instances stored in the memory, which are retrieved based on the highest blending value. IBLT is explained by three slots in an instance i.e. *Situation *(*S*),* Decision *(*D*) and* Utility *(*U*) to term it as SDUs^23,24^. It contributes in gaining insight about decision making processes in a dynamic neuropsychological task^24^ and may be proven beneficial for determining association between dystrophin isoforms and cognitive deficits. Stroop color and word task (SCWT) was selected for IBLT based modeling of DMD data. Stroop task is a popular neuropsychological battery for the assessment of executive functioning, cognitive control and response inhibitions^25^. An intriguingly long studied aspect, called Stroop effect, was chosen in the initial computational models to observe interference and its resolution in the subjects^26^. Interference resolution is one of the functions of cognitive control which moderates the execution of naming sequences relevant to the task to function against interference and distraction. Stroop effect was also previously modeled using artificial neural networking approach to obtain the function of prefrontal circuits during the stroop performance^27^. Instead of interference component, learned strategic control or interference resolution was assessed by Adaptive Control of Thought-Rational (ACT-R) cognitive architecture^28^. Previous studies have considered working memory maintenance, a crucial element in executive control system which influences performance of cognitive domains^29^. Hence, SCWT provides an opportunity to understand the phenomenon of cognitive inhibition as well as association to working memory process through cognitive control mechanism.

Therefore, we aimed to obtain the neuropsychological profile of Indian DMD subjects based on mutation location and to probe the cognitive domain based on a novel IBLT model.

## Results

### Participant information

**Figure 1:**
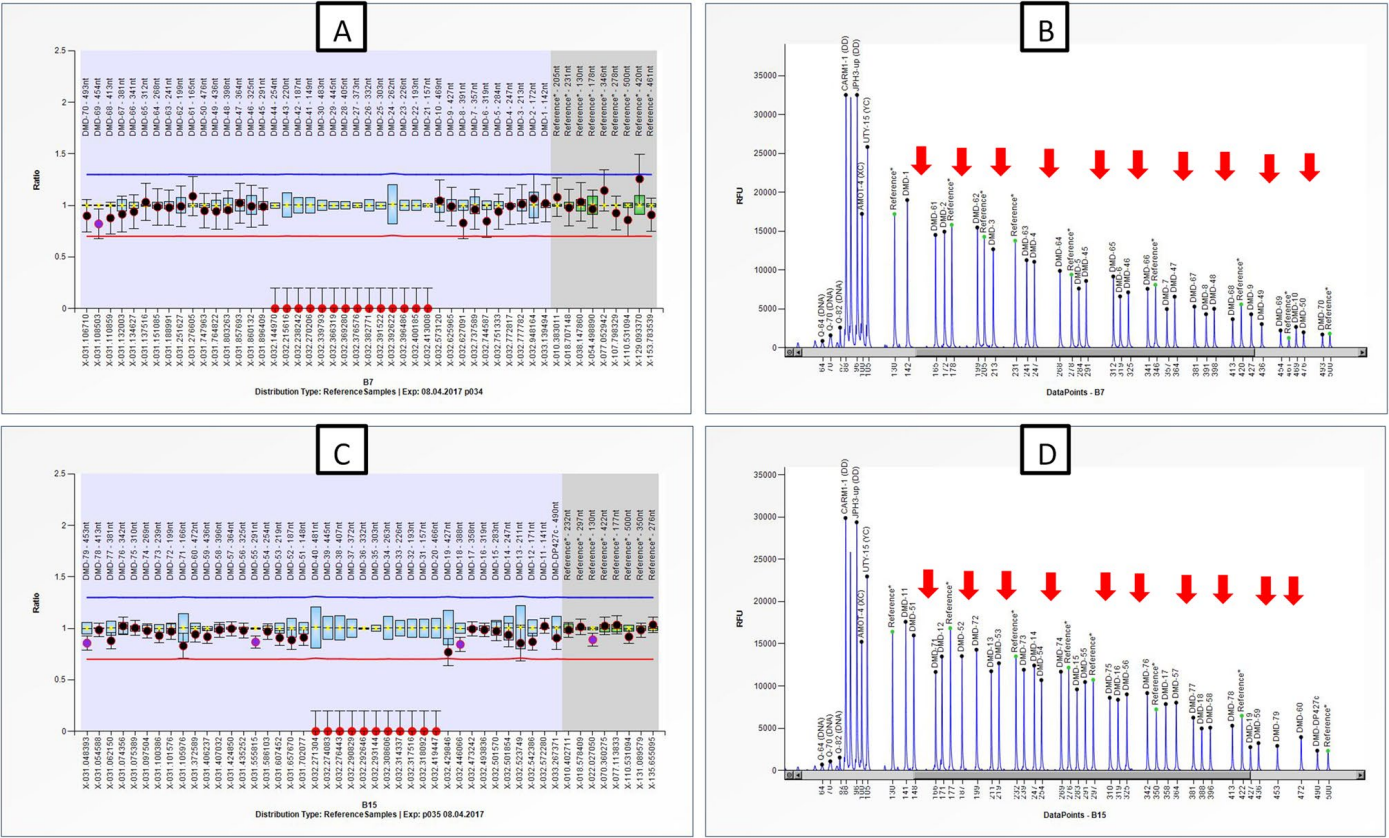
Figure represents ratio chart obtained through coffalyser.NET showing profile of DMD subject with long stretch deletion between exon 20 and 44. (**A**, **B**) Ratio chart and electropherogram depicting exonic deletions from exon 41 to 44 and exon 21 to 30 covered by P034 probe-mix. (**C**, **D**) Ratio chart and electropherogram depicting deletions from exon 31 to 40 and exon 20 covered by P035 probe-mix. P034 and P035 covers probes of all 79 exons of the *DMD* gene. Ratio between 0.70 and 1.30 was considered in the normal range while ratio of 0.00 was considered as deletion (depicted in red dots in ratio chart and red arrows in the electropherogram).

A total of 84 DMD cases with > 6 years of age, were subjected to MLPA based screening of copy number variations (CNVs). Spectrum of CNVs in our DMD cohort has been reported previously^30^. MLPA analysis revealed CNVs i.e. deletion/duplication in 67/84 DMD cases. Out of these 67 cases, 53 had mutation at region predicted to affect Dp140 isoform and 14 DMD cases had CNVs not affecting Dp140. MLPA based detection did not reveal CNVs in 17/84 DMD cases, indicating possibility of point mutation. Representative electropherograms depicting multiple exon deletions of dystrophin gene and normal pattern in controls have been illustrated in the Fig. 1. Point mutations or breakpoints could not be confirmed by Sanger/next generation sequencing.

**Figure 2:**
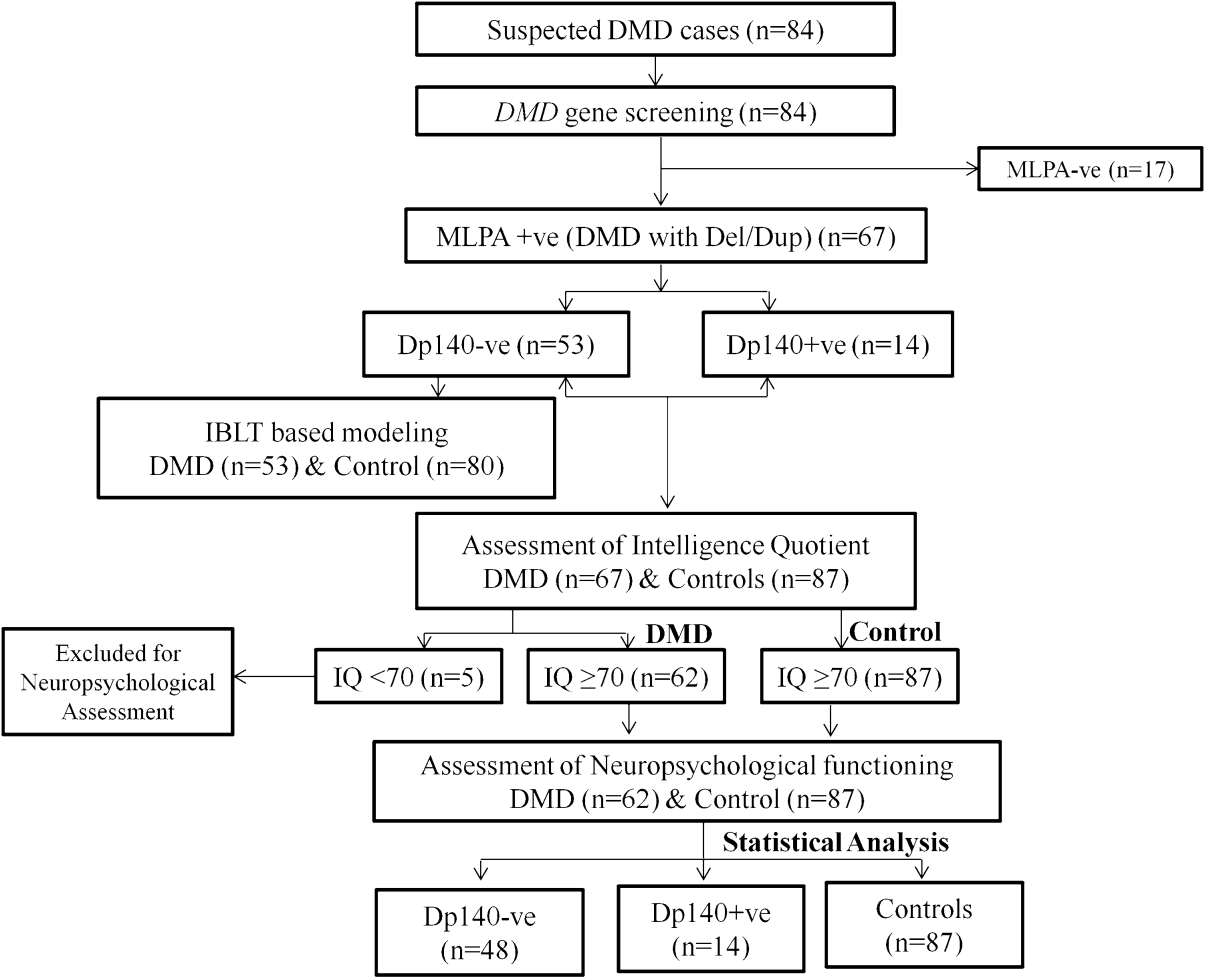
Flow chart of the study.

Cognitive and neuropsychological profiles were analysed for MLPA + ve cases (n = 67) along with age, sex and education matched control subjects (n = 87). Among 67 DMD cases, 53 cases were Dp140 − ve and 14 were Dp140 + ve. Out of 53 Dp140 − ve cases, five had intellectual disability and were not carried forward for neuropsychological (domain wise) investigation. Therefore, comprehensive neuropsychological data analysis was carried out in 62 DMD (Dp140 − ve, n = 48; Dp140 + ve, n = 14) cases. In view of educational dropping in our DMD cases, dropped out controls were also recruited. Hence, we used raw test scores for analysis, as the data was compared with matched control group. Computational modeling was carried out in all the Dp140 − ve cases (n = 53) and controls (80). Experimental chart has been provided in Fig. 2.

### General intellectual functioning in DMD

General intellectual abilities of DMD subjects with age group > 6 years were compared to the control group. Both the age groups were comparable with respect to age (p = 0.239) and education (p = 0.844). General intellectual functioning was categorized based on ICD-10 guidelines^31^. Out of 67 DMD cases, 49 (73%) had adequate intelligence (IQ > 84) and 13 (19%) demonstrated borderline intelligence (IQ = 70–84). Only 5 cases demonstrated intellectual disability (IQ < 70) among which 1(1%) had moderate and four (6%) had mild intellectual disability. DMD cases with intellectual disability were found to be associated with Dp140 − ve category with deletions involving exon 44/45, as depicted in Table 1. Moreover, among the cases with borderline intelligence (IQ 70–84), 69% belonged to Dp140 − ve category (predicted to have affected Dp140 isoform) and 31% to the Dp140 + ve category (predicted to have normal expression of Dp140 isoform). This data indicate impact of Dp140 and its genomic location in the manifestation of intellectual disability in DMD. The number of Dp140 − ve and Dp140 + ve cases varied in our cohort resulting in the group size difference. This differences have also been reported in earlier studies^30^.

**Table 1:** Details of DMD cases with intellectual disability.

S. no.	IQ	Severity of mental retardation	Mutation	Predicted loss of short Dystrophin isoform^a^
Case-1	42	Moderate	Del Exon 45–52	Dp140
Case-2	55	Mild	Del Exon 45–52	Dp140
Case-3	57	Mild	Del Exon 45–49	Dp140
Case-4	65	Mild	Del Exon 45–52	Dp140
Case-5	66	Mild	Del Exon 20–44	Dp140

^a^Transcription start site was not confirmed.

Analysis of cognitive profile between Dp140 + ve (n = 14) and Dp140 − ve (n = 48) group was performed using suitable statistical test. DMD Dp140 − ve group performed better in the information subset of MISIC (t = − 3.632, p = 0.001). However, compared to the control group, Dp140 + ve and Dp140 − ve groups revealed statistically significant reduction in all measures of verbal and performance subsets as presented in Table 2.

**Table 2:** Comparison of general intelligence between DMD-proximal, DMD-distal and control group using analysis of variance (ANOVA).

Cognitive domain and neuropsychological battery	Neuropsychological battery variables	DMD-proximal Mean (SD)	DMD-distil Mean (SD)	Control Mean (SD)	F value	p value	Multiple comparison^a^		
							p value Prox vs distal	p value Control vs prox	p value Control vs distal
Verbal intelligence Performance intelligence General intelligence DMD distal (n = 48) DMD Pro-(n = 14) Control (n = 87)	Information	79 (7.92)	92 (17.82)	106 (16.30)	21.610	< 0.001	0.001	< 0.001	< 0.001
	Comprehension	82 (11.56)	79 (25.76)	110 (20.56)	33.594	< 0.001	0.543	< 0.001	< 0.001
	Arithmetic	84 (11.95)	82 (17.69)	105 (15.36)	34.238	< 0.001	0.620	< 0.001	< 0.001
	Digit span	85 (12.52)	85 (12.47)	95 (16.59)	7.980	0.001	0.944	< 0.001	0.019
	Similarity	89 (17.83)	97 (27.37)	121 (19.24)	1.286	0.286	0.281	< 0.001	< 0.001
	VIQ	85 (10.87)	88 (15.05)	107 (13.69)	37.374	< 0.001	0.410	< 0.001	< 0.001
	Picture completion	75 (24.71)	75 (24.13)	97 (16.10)	20.077	< 0.001	0.992	< 0.001	0.007
	Block designing	102 (20.73)	88 (37.08)	113 (20.89)	11.867	< 0.001	0.120	0.001	< 0.001
	Coding	76 (38.01)	85 (37.21)	108 (22.48)	12.358	< 0.001	0.503	0.017	0.014
	Maze	85 (52.86)	104 (39.97)	121 (14.67)	10.546	< 0.001	0.264	< 0.001	0.038
	PIQ	95 (11.79)	94 (17.98)	110 (12.62)	21.717	< 0.001	0.803	< 0.001	< 0.001
	IQ	90 (10.75)	89 (19.94)	108 (11.16)	37.259	< 0.001	0.864	< 0.001	< 0.001
Factor indexes	VCI	214 (76.45)	210 (110.48)	313 (97.15)	21.610	< 0.001	0.887	< 0.001	< 0.001
	WMI	151 (55.15)	140 (66.93)	187 (57.13)	33.594	< 0.001	0.528	< 0.001	0.037
	PRI	162 (60.67)	126 (80.99)	191 (67.33)	34.238	< 0.001	0.072	< 0.001	< 0.001

^a^Multiple comparison correction: Bonferroni.

### Specific cognitive abilities

#### Comparison of verbal memory between control, Dp140 + ve and Dp140 − ve groups

DMD subjects with intellectual disabilities were excluded for assessment of specific cognitive abilities. Comparison of RAVLT performance between Dp140 + ve and Dp140 − ve groups did not show any significant differences as seen in Table 3. However, when compared to the control group, proximal group (DP140 + ve) performed poor in RAVLT immediate recall (p = 0.029), serial positioning variables recency T-1 (p = 0.003), and combined middle (p = 0.047) and recency (p = 0.023) scores. Performance in the RAVLT Trial 1 was marginally affected (p = 0.054). However, when compared to performance of proximal DMD group with control, distal DMD group performed worse in Trial-1 (p = 0.048), Trial-5 (p = 0.024), learning capacity (p = 0.046), immediate recall (p = 0.031), delayed recall (p = 0.012), commission (p = 0.018), long term percent retention (LTPR; p = 0.016), RAVLT-Memory Efficiency Index (MEI; p = 0.007). We did not find any statistical significance in other RAVLT measures which are depicted in the Table 3. Overall, Dp140 + ve subjects were able to perform similar to the control group in 16/20 (80%) RAVLT measures, whereas Dp140 − ve subjects were able to perform similar in 10/20 (50%) RAVLT measures.

**Table 3:** Comparison of RAVLT performance in DMD cases with proximal and Distal Mutations and control subjects.

Cognitive domain and neuropsychological battery	Neuropsychological battery variables	Proximal Mean (SD)	Distal Mean (SD)	Control Mean (SD)	Proximal Mean rank	Distal Mean rank	Control Mean rank	Chi-Square	*p*-value	*p*-value (multiple comparison^a^)		
										Prox. vs distal	Control vs prox	Control vs distal
**RAVLT** Verbal learning Working memory Short term verbal memory Long term verbal memory	RAVLT-trial 1	6.00 (2.69)	6.73 (2.48)	7.48 (1.91)	60.21	70.15	85.38	6.307	0.043	0.424	0.054	0.048
	RAVLT-trial 5	11.29 (3.17)	11.38 (3.18)	12.71 (2.22)	63.86	68.24	85.93	6.780	0.034	0.843	0.070	0.024
	RAVLT-learning capacity	47.29 (10.62)	47.82 (13.56)	52.89 (9.54)	61.96	67.72	83.62	5.806	0.055	0.772	0.073	0.046
	RAVLT-IR	9.79 (3.02)	10.15 (3.74)	11.64 (2.74)	59.21	69.05	86.19	7.572	0.023	0.554	0.029	0.031
	RAVLT-DR	9.93 (2.92)	9.81 (3.48)	11.38 (2.89)	64.54	66.75	86.69	7.954	0.019	0.987	0.066	0.012
	RAVLT-hits	14.00 (1.47)	14.19 (1.81)	14.66 (0.69)	66.07	70.45	80.56	3.905	0.142	0.664	0.139	0.098
	Omission	1.00 (1.47)	0.83 (1.81)	0.33 (0.65)	86.00	81.84	71.28	4.168	0.124	0.679	0.132	0.084
	Commission	0.29 (0.47)	1.27 (2.99)	0.39 (0.95)	72.93	86.07	71.05	5.812	0.055	0.230	0.807	0.018
	LTPR	97.17 (44.94)	85.38 (20.57)	89.55 (18.57)	79.31	64.53	83.02	5.784	0.055	0.312	0.807	0.016
**RAVLT** Serial positioning effect Working memory	Primacy T1	2.50 (1.56)	2.37 (1.27)	2.70 (1.18)	73.93	71.82	82.25	2.020	0.364	0.904	0.522	0.166
	Middle-T1	1.71 (1.59)	2.21 (1.16)	2.21 (1.04)	60.71	78.63	80.35	2.499	0.287	0.166	0.119	0.814
	Recency-T1	1.64 (0.84)	2.06 (1.24)	2.58 (1.12)	52.32	68.30	87.71	11.854	0.003	0.297	0.003	0.012
	Primacy-total	17.79 (3.40)	17.17 (5.21)	19.09 (3.67)	67.50	69.52	84.61	4.580	0.101	0.981	0.151	0.060
	Middle-total	13.36 (5.72)	15.25 (4.65)	16.52 (4.53)	58.14	72.14	84.54	5.547	0.062	0.257	0.047	0.107
	Recency-total	15.29 (2.84)	15.46 (4.95)	17.44 (3.77)	58.61	69.26	86.16	7.574	0.023	0.571	0.023	0.035
**RAVLT** Susceptibility to interferences	Proactive interference	0.92 (0.52)	0.84 (0.36)	0.88 (0.29)	73.15	71.98	79.61	1.057	0.590	0.900	0.604	0.329
	Retroactive interference	0.96 (0.54)	0.88 (0.22)	0.92 (0.16)	65.46	72.54	80.43	1.979	0.372	0.578	0.256	0.298
	Forgetting speed	1.07 (0.31)	0.97 (0.18)	0.98 (0.17)	84.27	70.58	77.86	1.433	0.489	0.356	0.579	0.329
	RAVLT MEI	1.95 (0.23)	1.80 (0.44)	2.02 (0.25)	70.73	63.48	84.90	7.887	0.019	0.489	0.237	0.007

*RAVLT* Ray Auditory verbal Learning Test, *T1* Trial 1, *IR* immediate recall, *DR* delayed recall, *LTPR* long term percent retention, *RAVLT-MEI* RAVLT memory efficiency index.

^a^DMD distal (n = 48), DMD Pro-(n = 14), control (n = 87).

#### Comparison of SCWT measures between control, Dp140 + ve and Dp140 − ve groups

No statistically significant difference was found between Dp140 + ve and Dp140 − ve group. However, Compared to control group, Dp140 + ve group showed difference only in stroop-color reading task. Dp140 − ve group showed differences in all measures of SCWT (see Table 4).

**Table 4:** Comparison of stroop color and word task in DMD cases with proximal and distal mutation and controls.

Cognitive domain and neuropsychological battery	Neuropsychological battery variables	Proximal Mean (SD)	Distal Mean (SD)	Control Mean (SD)	ANOVA F-value	ANOVA p-value	Prox vs Dis t-value	Prox vs Dis p-value	Control vs Prox t-value	Control vs Prox p-value	Control vs Dis t-value	Control vs Dis p-value
Executive functioning SCWT^a^ Cognitive flexibility Cognitive control Response inhibition Interference	STROOP-W	48.00 (20.52)	52.30 (20.07)	60.09 (13.4)	4.798	0.010	− 0.63	0.53	− 2.092	0.058	− 2.149	0.036
	STROOP-C	37.25 (15.33)	38.46 (13.71)	46.79 (13.9)	8.405	0.000	− 0.24	0.81	− 3.493	0.003	− 3.318	0.002
	STROOP-CW	20.25 (7.35)	24.49 (9.44)	28.34 (9.8)	6.071	0.003	− 1.61	0.12	− 1.091	0.295	− 2.131	0.037
	STROOP effect 1	14.75 (10.55)	14.11 (8.51)	18.22 (9.1)	3.194	0.044	0.19	0.85	− 0.669	0.515	− 2.424	0.018
	STROOP effect 2	0.45 (0.15)	0.49 (0.13)	0.48 (8.2)	0.272	0.762	− 0.67	0.51	− 0.451	0.659	0.056	0.956
	STROOP effect 3	0.57 (0.16)	0.65 (0.19)	0.60 (8.9)	1.672	0.192	− 1.39	0.18	− 1.004	0.336	1.572	0.121

*SCWT* Stroop Color and word task-word, *color* color-word.

^a^DMD distal (n = 48), DMD Pro-(n = 14), control (n = 80).

#### Comparison of digit span measures between control, Dp140 + ve and Dp140 − ve groups

Assessment of short term memory, working memory and attention fraction revealed no difference between proximal (Dp140 + ve) and distal (Dp140 − ve) mutation group. Compared to control group Dp140 + ve as well as Dp140 − ve group demonstrated significant differences in digit span forward (DSF) and digit span backward (DSB) task (DSF: χ^2^ = 8.342, *p* = 0.015; DSB*:* χ^2^ = 11.103, p = 0.004) as depicted in Table 4. Attention fraction in the proximal group was comparable to the control group (p = 0.167), whereas distal group demonstrated significant difference compared to control group (p = 0.040). Statistical analyses of variables are depicted in Table 5.

**Table 5:** Comparison of digit span task in DMD cases with proximal and distal mutations with control group.

Cognitive domain and neuropsychological battery	Neuropsychological battery variables	Proximal Mean (SD)	Distal Mean (SD)	Control Mean (SD)	Proximal Mean rank	Distal Mean rank	Control Mean rank	Chi square	*p*-value	Multiple comparision *p* value		
										Prox vs distal	Control vs Prox	Control vs distal
**DIGIT span test**^a^ Short term memory working memory	DSF	4.73 (1.19)	4.94 (1.21)	5.52 (1.21)	53.68	62.09	79.85	8.342	0.015	0.499	0.046	0.015
	DSB	2.64 (1.29)	2.66 (1.84)	3.59 (1.26)	53.05	59.87	81.16	11.103	0.004	0.823	0.018	0.004
	Attention fraction	0.32 (0.25)	0.40 (0.36)	0.23 (0.18)	82.32	80.71	65.12	5.158	0.076	0.992	0.167	0.040

^a^Digit span test: DMD distal (n = 48), DMD Pro-(n = 14), control (n = 87).

### Model results on stroop color and word task (SCWT)

**Figure 3:**
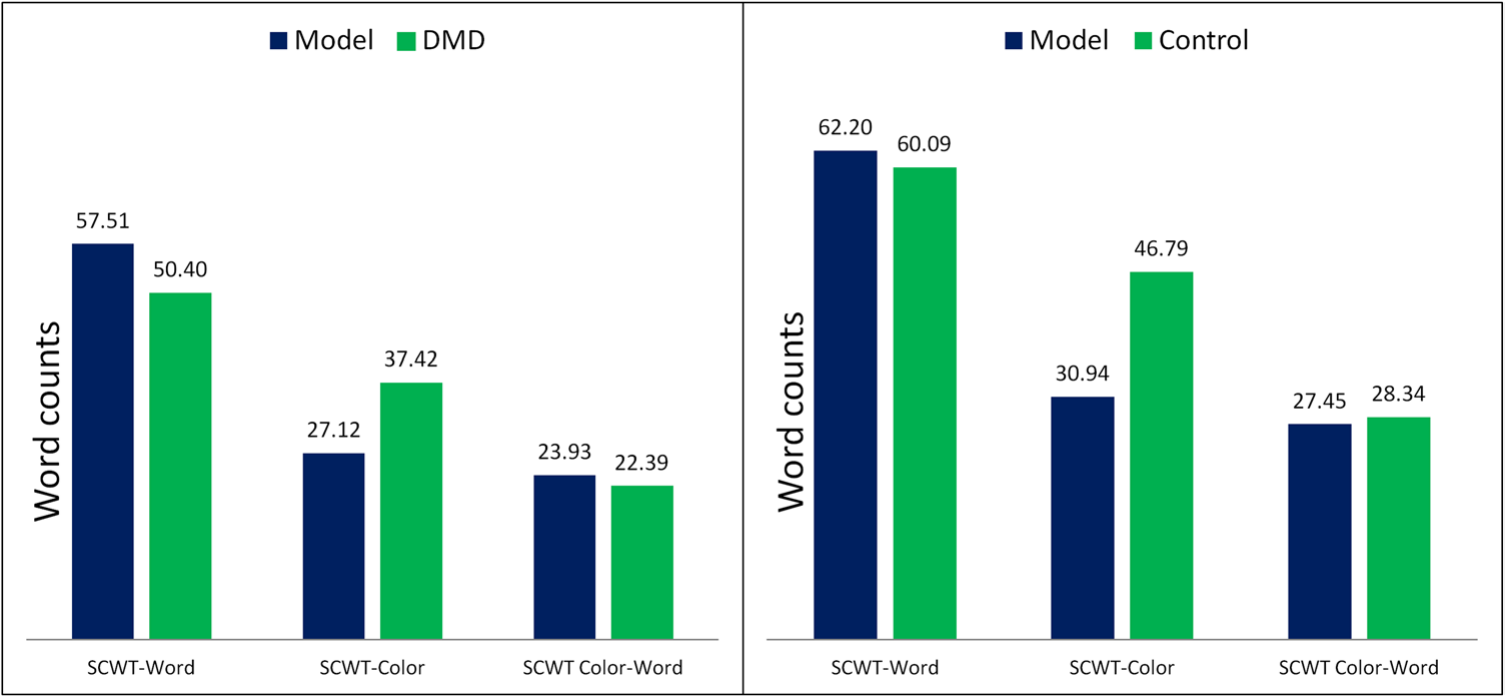
IBLT based computational modeling in DMD (n = 53) and control (n = 80) groups.

**Figure 4:**
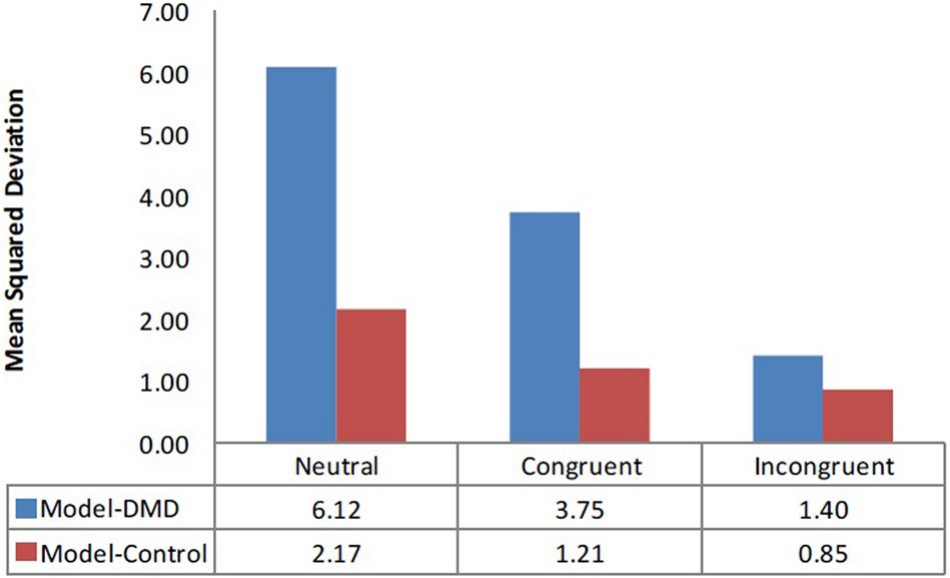
Mean Square Deviation (MSD) in the Model-DMD (n = 53) and Model-Control (n = 80) show the reduced MSD in the Model-Control than the Model-DMD. Controls performed better than the DMD group.

It was found that memory decay in control group was more compared to DMD group. The control group paid more attention (d = 5) to recent information compared to DMD group (see Table 6). Both groups showed low variability (low noise value “s”) in choice during different trials of SCWT. Reaction time represented by “f value” for control group was found to be reduced as compared to DMD group. According to the equation of RT, reaction time for experiment group is lower as compared to control group (Fig. 3). The mean square deviations (MSDs) obtained for DMD group are higher as compared to control group. Therefore, the IBL model shows poor performance for DMD group in comparison to the control group (Fig. 4).

**Table 6:** Computational modeling results representing modeling values.

IBLT based computational modeling	Latency factor (F)	Reaction time (f)	Decay value (d)	Noise value (s)
Normal range	0–1	0–1	0–10	0–10
Model vs DMD (n = 53)	1	0.005	4	2
Model vs control (n = 80)	1	0.003	5	2

## Discussion

Duchenne Muscular Dystrophy (DMD) is a fatal genetic disorder with variable degrees of intellectual deficits in one third of those afflicted involving multiple cognitive domains with heterogeneous cognitive strengths and weaknesses. Cognitive impairment is a multifactor phenotype governed by interactions of various genes with interplay of socioeconomic contexts^32,33^. Moreover, cross-cultural variations may impact the data obtained in the Indian sub-population which necessitated an independent study to assess the cognitive strengths and weaknesses across the cognitive domains. The study was important for exploring management strategies to obtain a better quality of life for DMD patients.

In our cohort, 26% of DMD subjects presented variable degrees of cognitive impairment. Only 7% of DMD subjects were found to have intellectual disability. Surprisingly, all DMD cases with intellectual disability were found to be associated with deletions in exon 44/45 predicted to affect Dp140 isoform. Moreover, 69% cases with borderline intelligence belonged to Dp140 − ve category. This data indicates crucial impact of Dp140 and its genomic location in the manifestation of intellectual disability in DMD. Dp140 − ve group lacking crucial Dp140 brain isoform showed differences in the RAVLT Trial 1 indicating crucial role of Dp140 isoform in maintaining working memory, whereas Dp140 + ve group was found to hold working memory functions similar to the normal subjects (Table 3). It indicated the crucial role of exon 44/45 DMD gene due to its involvement in the expression of Dp140 isoform. Similar verbal working memory alterations were found in the DMD 6–10 years age group and not in higher age group (see Supplementary information). It is crucial to discuss here that distal Dp140 isoform is mainly expressed during fetal development^34–36^ reflecting its role in altered working memory during early ages. Dp140 + ve (Proximal mutation, Dp140 intact) group showed learning equivalent to the control group in all RAVLT acquisition trials whereas Dp140 − ve group showed similar word acquisition only till trial 4, indicating saturation in the acquisition of verbal information. It indicates the role of DMD distal mutation (Dp140 affected) in the altered learning capacity of DMD. DMD 6–10 years of age group also showed poor learning trends in each acquisition trials with inadequate learning capacity. Saturation in the learning performance also indicates poor availability of neural resources to support higher level cognitive processing consistent with the neural integrity as suggested by Dixon et al.^37^. The distal mutation location in DMD has previously been associated to severe intellectual deficits^5,19,35,38^.

Poor performance in recalling list B indicates susceptibility to interference from previously acquired memory. This might be responsible for hampering the acquisition of new memory. It also indicates the failure of brain processes to shift flexibly from one set of information to another. However, retroactive as well as proactive interferences in DMD, were found to be similar to the control group (Table 3) indicating normal susceptibility to interferences due to previously acquired or newly acquired information. Presence or absence of Dp140 isoform did not affect the ability of DMD to respond to interferences. Proactive interference also determines the health of “hippocampal CA3” and Dentate gyrus region of brain^39^. However, the effect of newly learned information on the recall of previous information, also called retroactive interference, indicated a normal hippocampal CA1 region^40^. Normal susceptibility to interferences further validated deficits in the verbal working memory axis through List B recalling. As compared to control group, Dp140 + ve DMD group showed difference only in the immediate verbal memory, whereas Dp140 − ve group showed alterations in short as well as long term verbal memory. It was further validated by LTPR scores. Dp140 + ve DMD group preserved the percent retention whereas Dp140 − ve group lost the retention capabilities. We combined all measures of RAVLT to estimate RAVLT memory efficiency index (RAVLT-MEI) based on the procedures adopted in previous study^41^ which combined measures of encoding and retention. Ricci et al. reported the cut off range of 1.2 and 1.9 to differentiate the patients with Alzheimers Disease/behavioural variant Fronto temporal dementia (bvFTD) and bvFTD/Normal and controls respectively in order to evaluate diagnostic impact of this factor index. DMD group lacking Dp140 isoform showed RAVLT-MEI value equivalent to the patients with behavioural variant Fronto temporal dementia indicating a lowered verbal memory efficiency index. However, DMD group with preserved Dp140 isoform showed RAVLT-MEI cut off value similar to the value of control group. In a normal condition, serial positioning effect necessitates recalling first and last words and items from a list. Recently learnt information remains in the working memory and initially learnt items are considered to be a part of long term memory, hence, middle items did not get the benefit of both memories and are encoded poorly. DMD groups (including Dp140 + ve and Dp140 − ve) were equally affected in the acquisition of recently learnt words which formed recency scores [obtained in trial-1 and combined trials (T1–T5)] indicating deficits of immediate working memory axis. Interestingly, absence of DP140 isoform did not affect the middle order of the list whereas Dp140 + ve group exhibited the changes in the acquisition of middle order list.

Digit span test was used to measure attention, short term memory (DSF) and working memory (DSB). DS-Forward and DS-Backward performance were altered and reflected poor short term memory and working memory respectively, irrespective to the dystrophin mutation location. Alterations in the DSF and DSB scores were obtained in the DMD age group 6–10. DSB task attention fraction indicated requirement of attentional loop in completing tasks which involves switching of short term memory processes to working memory processes in brain. Dp140 lacking DMD group showed altered utilization of attention fraction. In our study, working memory deficits were indicated by RAVLT Trial 1, arithmetic subtest, digit span backward task, recency effect and working memory index. Deficit in the frontal lobe functions such as organization and manipulations of the information, which are distinct from the short term storage, may be probable site of dysfunction in DMD. Moreover, RAVLT commission score suggested that working memory capacity seems to be governed by cluttering of irrelevant content which reduces the effective capacity of relevant contents, suggesting processes similar to aging brain. Dp140 − ve but not Dp140 + ve group showed changes in the commission subset which indicates that lack of Dp140 isoform positively modulates the working memory through cluttering of irrelevant content. Based on baddeley’s model, the coordination of resources is the crucial element of working memory for which memory acts as a potential demand^42^. However, multiple working memory exposure (rehearsal) of information is required for complex cognitive performance including short and long term memory. DMD subject’s (especially Dp140 − ve) multiple exposure to the information through RAVLT trials enabled them to maintain the information till 4th trial. It indicated the crucial role of Dp140 isoform in acquisition, encoding and retrieval of verbal memory. Moreover, inability of the DMD groups (especially Dp140 − ve) to use the working memory processes may have influenced the information processing leading to alterations in higher order cognitive functions including verbal fluency, cognitive control, visuo-spatial manipulation^43,44^. Poor stroop color word task (SCWT)-Word (W) performance corresponds to personnel tempo, speech motor problems and learning disabilities. Alterations reflect the health of primary visual cortex since it is responsible for the spatial selectiveness and colour perception^45,46^. Dp140 + ve (Intact Dp140 expression) mutation group demonstrated an intact performance in SCWT word and color word task compared to control group. Dp140 isoform protected group was also found to have a normal susceptibility to stroop interference. However, DMD group lacking DP140 isoform (DP140 − ve) showed alterations in all measures of SCWT tasks. Altered brain interference tendencies of the DMD group over an alternative stimulus provides the functionality of particular domain which regulates executive control of response inhibition.* Anterior cingulate cortex* is reported to be robustly activated on attentional processing of selecting appropriate and suppressing inappropriate verbal responses through stroop color word task^47^. DMD early age group (6–10 year) was found to have the susceptibility to stroop interference.

Age wise analysis of DMD cognitive abilities showed interesting trend of deficits in the age group of 6–10 years indicating the role of early life education and neuropsychologcal rehabilitation in the DMD subjects. Moreover, all DMD cases below 6 years of age also demonstrated mental retardation or borderline intelligence. Studies have shown that susceptibility to interferences is more in the early ages and there is age dependent increase in the resolutions of interferences^48,49^. This phenomenon develops human brain to be less prone to interferences. Age wise analysis of DMD cases indicates a normal trend of interference resolution in the cognitive flexibility and attention. Though working memory measures also showed a normal trend in later developmental ages, in early ages, it potentially contributes in the overall deficits in various cognitive domains. We have earlier reported a non-progressive neuropsychological functioning in the DMD subjects. Therefore, we now believe, that the early stages of DMD are suitable interventional age in the DMD kids.

Stroop task explores functionality, due to the congruency between the word and the ink color, which varies in presence of conflicts between written word spell and ink color. It produces latency and errors, also known as Stroop effect. Studies, explaining the insufficient cognitive control on the Stroop interference, describe automatization of word reading as crucial element in the cognitive processing, even under the influence of color-naming instructions^50^. Other studies demonstrated response compatibility^51^, speed of processing^52^ and differential translation requirements^53^ as an explanation to Stroop effect. However, inhibition in the Stroop task account for a negative situation also called negative priming and describes weakness.

Previous studies have also reported working memory impairment in DMD subjects^19,35,54,55^. However, its association with Dp140 isoform has not yet been validated through modeling strategies. In our study, IBLT-ACT-R based cognitive computational modeling of stroop color and word task revealed non-reliance of DMD subjects towards recency (recently learned information), which indicated poor working memory capacity. The variability in choice was similar for both DMD and control groups. DMD group also showed lower activation of instance retrieval, resulted in increased response time. It also showed that the control group was found to be more attentive compared to DMD group. Involvement of working memory component inter alia multiple cognitive domains especially phonological and attention circuit in DMD patients necessitated development of analytic and interventional computational modeling of multi-component model of working memory. IBL based computational modeling indicated reduction of the working memory capacity in the DMD subjects who were also less attentive in capturing recent information (Table 5). Smaller “s value” is believed to be a multiplier into activation which indicated lesser retrieval of instances from the memory. Activation for the instances has been found to be poorer in the DMD group. Lower “d value”, indicated that the DMD group exhibited less on recency and more on primacy. These results were also obtained from the assessment of RAVLT serial positioning recency effect. Elapsed retrieval time also indicated that DMD group took more time to recall the instances or experiences from memory. DMD group’s non-reliance on recency also indicated that the closest instances or experiences are taking longer retrieval time from the memory, since recently learned information have less impact on the memory and decision making in DMD. It represents a lesser capacity of working memory in DMD. However, DMD groups reliance on primacy suggested scope of improvements in the memory processes, if working memory is targeted through continuous rehearsal and training.

The current study, for the first time utilized novel modeling strategies to delineate the cause of cognitive phenotypes in the DMD. Though the appropriate statistical tests were used in the study, uneven distributions of DMD cases with proximal and distal mutation location limit us and warrant larger sample size in proximal group. Due to limited number of cases with proximal mutations computational modelling could not be performed in this group.

## Conclusion

Dp140 isoform and working memory axis play crucial role in the development of altered but non progressive cognitive and neuropsychological profile in DMD. IBLT based computational model validated Dp140 isoform associated alterations in the working memory axis. Neuropsychological rehabilitation at early age may be devised to focus on working memory training by various interventional approaches including computational neurobics, yogic and meditation regimen. Improving cognitive fitness in DMD would improve the quality of life.

## Methodology

### Participants

A total of 84 DMD subjects were enrolled after obtaining informed assent from the children and informed consent from the parent or legal guardian according to the guidelines of Institutional Ethics Committee (IEC) of the Postgraduate Institute of Medical Education and Research, Chandigarh (Vide INT/IEC/2015/732). Written Informed consent was obtained from all participants and considered mandatory for participation in the study. Experimental protocols were approved by Dean Doctoral Committee and Doctoral Committee followed by IEC, PGIMER, Chandigarh vide (Vide INT/IEC/2015/732). Cognitive assessment was carried out in 84 DMD and control subjects. Inclusion criteria involved presence of *DMD* gene variants in the DMD subjects. An Intelligence quotient below 70 was chosen as an exclusion criteria for neuropsychological assessment as per ICD-10 guidelines.

### Inclusion criteria

- Dystrophin gene mutation
- Age > 6 years
- Informed assent and consent according to the standard ethical guidelines outlined by Institutional Ethical Committee (IEC) of PGIMER, Chandigarh.

### Exclusion criteria

- Co morbidities: Autism, epilepsy, autism spectrum disorder (ASD), obsessive–compulsive disorder (Only for neuropsychological assessment)
- Mutations in *DYSF, LMNA, CAV3, SMN, SMN, APP, PSEN* genes in the patients of *DMD* − ve variants.

### Multiplex ligation probe amplification (MLPA)

Genomic DNA was extracted from the lymphocytes or whole blood of DMD patients as per manufacturer (QIAamp DNA Blood Mini Kit, QIAGEN) guidelines. The DNA samples were coded as per GLP module and stored at – 20 °C. MLPA probe sets, P034 and P035 (MRC-Holland, Amsterdam, the Netherlands) were used for detecting variations in the target region spanning 1–79 exons of *DMD* gene as reported previously^56^. Detailed procedure has been described in Supplementary information.

### DP140 + ve and DP140 − ve group

Based on the predicted absence or presence of Dp140 isoform of *DMD* gene, DMD participants were grouped as Dp140 + ve or Dp140 − ve. Dp140 + ve corresponds to the group of DMD subjects who carried deletion/duplications upstream from Dp140 promoter region i.e. Exon 44 as described earlier^5,18^. Dp140 − ve corresponds to group of DMD subjects who showed deletion/duplications downstream of Dp140 promoter region.

### Cognition and neuropsychological profiling

General intelligence was assessed using age appropriate standard tests to obtain verbal, performance and full-scale IQ. Subjects with IQ > 70 were considered for assessment of specific cognitive domains. Digit span backword task, a component of verbal Subset, was considered as a measure for working memory. Rey Auditory Verbal Learning Test (RAVLT) was administered to assess working memory, verbal learning and memory. RAVLT trial 1 score was also considered as a measure for working memory. Stroop Color and Word Test (SCWT) was performed to measure the response inhibition and cognitive control. Detailed methodology has been provided in the Supplementary information.

### Computational cognitive modeling

We used a novel approach of computational modeling of Stroop task based on ACT-R cognitive architecture’s partial matching process^57^. This account is different from strategy-based account, which has been reported in literature^58–60^**.** The partial-matching process assumes that dissimilarity between memory instances and stimuli in the environment create a penalty in instance’s activations, making it difficult to retrieve these dissimilar instances. An Instance-Based Learning (IBL) model, based upon the ACT-R cognitive architecture, was implemented to model human decisions in SCWT. An instance in IBL model for stroop task consists of Color, Word, and Outcome. A manually conducted Stroop task was converted to a computer matrix. Three sheets were prepared to be read by computer model in order to obtain the response time: Neutral, incongruent, congruent. Forty-five seconds were provided for each sheet. We used incongruent stroop stimulus which consist of word spell and ink color to be different to obtain scores of stroop interference. Instances created in the previous two sheets were used to obtain the response time. The partial matching mechanism proposed in IBLT was used as a working memory capacity indicator, since it determines the number of SDUs used in the recognition and judgment processes. Deviation from the actual word read was considered to be a measure of the distance between two words. Deviations were considered punishment which helped the model to learn and to have a good decision. In IBLT, the activation of an instance ‘i’ in memory is defined using the ACT-R architecture’s activation equation:

1 $$A_{i} = B_{i } + \Sigma_{l = 1 }^{k } P_{l} \times M_{li } + \varepsilon_{i} .$$

In this equation, M_li_ represents the mismatch between the requested value and the retrieved value, which can vary between 0 (no mismatch, so no penalty) and − 1 (complete mismatch). P_l_ represents the penalty that is deducted from the activation in case of a complete mismatch. Furthermore ϵ_i_, is the noise value that is computed and added to an instance i’s activation at the time of its retrieval attempt from memory. The noise value is characterized by a parameter s. The noise is defined as:

$$\in_{i} = s{ }.{ }ln\left( {\frac{{1 - \gamma_{i,k,t} }}{{\gamma_{i,k,t} }}} \right),$$

where, η_i_ is a random draw from a uniform distribution bounded in [0, 1] for an instance i in memory. Bi is the base-level learning parameter and reflects the recency and frequency of the use of the ith instance since the time it was created, which is given by:

2 $${\text{B}}_{i} = ln\left( {\mathop \sum \limits_{{t_{i } \varepsilon \left\{ {1, \ldots ,t - 1} \right\}}} (t - t_{i} )^{ - d} } \right) .$$

The frequency effect is provided by t − 1, the number of retrieval of the ith instance from memory in the past. The recency effect is provided by t − t_i_, i.e., the event since the tth past retrieval of the ith instance (in Eq. 2, t denotes the current event number in the scenario). The d is the decay parameter and has a default value of 0.5 in the ACT-R architecture.

#### Retrieval time (RT)

The time that it takes the declarative module to respond to a request for a chunk, *i*, is determined by the activation that the chunk has been using this equation when the sub-symbolic computations are enabled:

$$RT = Fe - \left( {f \times Ai} \right).$$

RT is the time to retrieve the chunk in seconds, Ai is the activation of the chunk *i* which is being retrieved, F is the latency factor parameter, f is the latency exponent parameter.

The latency factor value, F, in the equation for retrieval time, was obtained. It was set as default value of 1. Appropriate values of d, s, f and F were calculated through trial and error method. Values represent memory of previous instances and attention capacity for recent events. Higher “d or decay value” represents the subjects’ inability to recall previous events and more attentiveness to the recent instances. s value represents noise value and indicates variability in the decision making. Range of d and s was from 0 to 10. Optimization of these values was obtained by “hit and trial” method. However, range of f and F was considered 0 to 1.0. Higher f and d value represent a reduced working memory capacity as one remembers only recent information. Fitness function was obtained by subtracting number of words by human subjects to the number of words by model.

### Statistical analysis

We used SPSS version 16 (SPSS Inc., Chicago, IL, USA) for statistical analysis. Kolmogorov–Smirnov (KS) test was performed to assess the normality of data set. For normally distributed data, a parametric one-way analysis of variance (ANOVA) was applied to compare the differences between multiple groups. multiple comparison correction was carried out by post-hoc analysis through bonferroni method. For comparing differences between two groups unpaired/independent student-t test was performed. Equal or unequal variance was checked through Welch’s correction method. For non-normal data, a non-parametric Mann–Whitney-U test was applied for testing hypothesis between two groups. Statistical assessment for more than two groups was performed by Kruskal–Wallis H test followed by bonferroni correction. Statistical significance was considered at p < 0.05.

## Supplementary information

Supplementary information. (PDF 207 kb)
