# Supplementary material for: Computational cognitive modeling and validation of Dp140 induced alteration of working memory in Duchenne Muscular Dystrophy
Source: Sci Rep. 2020 Jul 20;10:11989. doi: 10.1038/s41598-020-68381-9 (PMC7371893; doi:10.1038/s41598-020-68381-9)
Supplement: Supplementary file 1 — Supplementary information. (PDF 207 kb) [file 41598_2020_68381_MOESM1_ESM.pdf]

Supplementary info

Computational cognitive modeling and validation of Dp140 induced alteration of working memory in Duchenne Muscular Dystrophy

Rahul Tyagi[1], Palvi Aggarwal[2], Manju Mohanty[3], Varun Dutt[2], Akshay Anand[1]

Materials and Method

Multiplex Ligation Dependent Probe Amplification (MLPA)

DNA was extracted from the lymphocytes or whole blood of DMD patients as per manufacturer (Qiagen) guidelines. Deletions and Duplications in the *dmd* exons were detected by MLPA. The DNA samples were coded as per GLP module and stored at -20°C. MLPA probe sets, P034 and P035 were used for detecting mutation in the target region spanning 1-79 exons of Dystrophin gene. Briefly, DNA samples along with three reference samples were denatured at 95°C for 1 min, followed by incubation at 60°C for 16–20 h for hybridization reaction. Hybridized probes were ligated at 54°C for 15 min and 98°C for 5 min by using ligase buffer (Buffer A & Buffer B) and ligase enzyme (Salsa Ligase 65). Amplification conditions were used as mentioned: 95°C for 30 s, 60°C for 30 s, and 72°C for 60 s, followed by 20 min at 72°C and a pause at 15°C for 35 cycles. Amplified sample (0.8µl) was mixed with 8.6 µl HiDi formamide and 0.4 µl size standard (Liz 500, Rox 550, Rox 500). Samples were denatured for 3 min at 86 °C, followed by immediate cooling for 2 min at 4°C. Fragment separation was carried out in the ABI platform and raw files (.fsa) were analyzed by coaffalyser.NET software. Inter-laboratory validation of the MLPA analysis was also carried out in other laboratories. Capillary electrophoresis data was analysed by Coffalyser.NET software (MRC Holland) to obtain the dosage quotient (DQ). DQ between 0,70-1.30 was considered normal, DQ 0.00 homozygous deletion and DQ between 1.75-2.15 homozygous duplication. Variations in the MLPA data were normalized through inter and intra-sample normalization processes by Coffalyser.NET software with peaks of reference samples and reference probes respectively.

Malin's Intelligence Scale for Indian Children (MISIC):
Assessment of general intelligence was performed by MISIC, an Indian adaptation of "Wechsler Intelligence Scale for Children" (WISC) with age between 6 to 15 years and 11 months old. It consists of 6 subsets for assessing verbal intelligence and 5 subsets for performance intelligence.

Verbal Subsets:
Information subset is a measure of general knowledge based on acquired facts stored in the long term memory according to the cultural requirements. **Comprehension subset** assesses the development of social awareness, practical judgment, moral conscience, common sense and social maturity of a subject. **Arithmetic** subset measures the ability of problem solving and concentration of a subject. **Analogies and Similarities** measures the participant's ability of comparative reasoning. **Vocabulary** measures the verbal knowledge and concept formation of a subject. **Digit Span (DS) Test** is used to measure attention, short term memory and concentration of a subject. It consists of DS-forward (DSF) and DS-Backward (DSB) task. DSF measures "short-term auditory memory processes", whereas, DSB measures "working memory" of participants. We, therefore, calculated the difference between DSF and DSB in order to obtain the attention fraction from the digit span score and termed as "attention fraction". Raw scores were converted into age appropriate TQ by using the normative data. Average TQ values of the subsets form **verbal intelligence quotient (VIQ)**.
Performance Subsets:
Picture Completion test measures focusing attention, concept formation and scanning of a subject. **Block Designing** was performed to measure spatial problem-solving, visuo-constructional abilities, manipulative abilities, Visuo-motor coordination and fluid intelligence of a subject. **Coding** measures visuo-motor coordination and speed of a subject. **Maze** measures visuo-spatial planning ability of a subject**. Object Assembly measures** visual anticipation skills, visual-motor problem-solving and organizational abilities. Raw scores were converted into age appropriate TQ of individual subset by using the normative data. Average TQ value forms **performance intelligence quotient (PIQ).** Various subsets of MISIC were scored according to the instructions provided in the manual. Average score of VIQ and PIQ was obtained to form a **full scale intelligence quotient (IQ)**

CHILDREN >16 YEARS:
Verbal Adult Intelligence Scale (VAIS): This is a measure of verbal IQ. It consists of 4 subtests: a) Information b) Digit Span c) Arithmetic and d) Comprehension. The items of these subtests were developed on the basis of WAIS. It consists of four subsets, information, comprehension, arithmetic and digit span to form a VIQ score. Norms for 3 educational levels and 5 age groups separately for males and females have been provided (1).

Bhatia's short battery of performance test: It consist of Kohs block design (KBD) test and Alexander's pass along test (APA), were used to form the PIQ. Average score of VIQ and PIQ was obtained to form a full scale intelligence quotient **(IQ) (1).**

Rey Auditory Verbal Learning Test (RAVLT): It was performed to measure auditory learning and memory, immediate memory span, susceptibility to interference, serial positioning effect, recognition memory and verbal memory efficiency. In the present study, the adapted version for Indian population was used (2). In this test, List of 15 nouns (List A) was read aloud during 5 consecutive trials followed by the recall in each consecutive trials. In each trial interval of one second was maintained between presentations of two nouns. A subsequent list (list B) of 15 words were presented as an interference after List A (Trial 1-5) to be recalled. Subject was asked to recall the List A immediately after List B task. After 20 min subject was then instructed to recall list A again to access the long term verbal memory. Subjects were then presented with 30 words (15 -list A, 15 other) for assessing recognition memory (List A words). The errors in the form of omissions (from List A), commissions (non list A words) scores are noted down (3).

Number of words correctly recalled in each RAVLT trial as well as in immediate (after List B) and delayed recall (after 20 min.) task were used as scores. Learning capacity was assessed by summing the total list-A words recalled over five trials. Subject's susceptibility to the interference was obtained through proactive interference (PI), retroactive interference (RI). PI reveals negative effect of previously learned material in the acquisition or recall of new information (4). Serial positioning effect was assessed by obtaining primacy (First $1/3^{rd}$ of 15 words), middle (Middle $1/3^{rd}$ of 15 words) and recency (Last $1/3^{rd}$ of 15 words) scores of List A for all trials (5). RAVLT Memory efficiency Index (MEI) was obtained by using RAVLT components based on the previous study (6)**.** Formulas used to calculate various scores have been represented in **Table 2.**

Supplementary Table 1: Details of various factor scores obtained from RAVLT trials to measure components of verbal memory functioning

Factor index	Formula
RAVLT Memory Efficiency Index (RAVLT-MEI)	[(delayed recall A/15)/(RAVLT Trials 1–5/75)]+[(delayed recognition hits/15) – (false positive/total number of distracters)]
Proactive Interference (PI)	RAVLT List B/RAVLT trial 1
Retroactive Interference (RI)	RAVLT Immediate Recall/T5
Forgetting Speed (FS)	RAVLT Delayed Recall/ RAVLT Immediate Recall
Long Term Potential Retention (LTPR)	DR/T5*100
Primacy Total	Sum of the first $1/3^{rd}$ of words recalled over 5 trials of list A
Middle Total	Sum of the middle $1/3^{rd}$ of words recalled over 5 trials of list A
Recency Total	Sum of the last $1/3^{rd}$ of words recalled over 5 trials of list A

Stroop Color and Word Test (SCWT): Stroop test was used to assess response inhibition, selective attention, and cognitive flexibility (7, 8). Stroop color and word task consists of a 5 by 20 matrix of words representing three colors (Red, Blue, Green) each in three sheets. First sheet consisted of 100 (5*20) names of three colors (Red, Blue, Green) printed only in black color. Subjects were instructed to read the words down the column for 45 seconds. Second sheet consisted of 100 (5*20) XXXX symbols printed in three colors (Red, Blue, Green). Subjects were instructed to name the colors down the column for 45 seconds. Third sheet consisted of 100 (5*20) color names which were printed in another color (Red/Blue/ Green). E.g. "red" written word printed in Blue or Green color and so on. Subjects were instructed to name the color instead of reading the written words down the column for 45 seconds. Number of words read in each sheet was considered the score of a participant. The last task has an interference component because it requires the participant to override or inhibit a reading response. This test measures the ease with which a person can shift his or her perceptual set to conform to

changing demands and inhibit usual response from interfering with the unusual one. Interference component (also called stroop effect) calculation formula is presented in Table 2 adapted to previous studies (9).

Supplementary Table 2: Calculations used to obtain various stroop effects.

Stroop Effect	SCWT Color (Raw)-SCWT Color-word (Raw)
Stroop Effect 1	Color raw score (C)-Color word raw score (CW)

Results:

Effect of Age on the cognitive and neuropsychological profile

DMD and control subjects were categorized to three age groups of (6-10 years, 11 to 16 years and > 16 years) to understand the effect of age associated changes. DMD '6-10' years group showed significant differences in the general intellectual abilities when compared to the control '6-10' year group (VIQ: p=<0.001; PIQ: p=<0.001; IQ: p=<0.001. Similar results were obtained between DMD '11-16' years group and control '11-16' years group. However, DMD'>16' age group did not reveal significant differences in comparison to the age matched control group. See supplementary data for details.

Age wise analysis of the verbal memory revealed that the DMD '6-10' years group showed significantly poor auditory verbal performance in RAVLT trial 1 (p=<0.001), Trial 2 (p=0.018), Trial 3 (p=0.026) and trial 5 (p=0.024), Learning capacity (p=0.005), delayed recall (p=0.006) and LTPR (p=0.01) in comparison to the control '6-10' years group. Similarly, among the serial positioning effect, only recency effect was significantly different (p<0.001) among DMD of this age group as compared to the control group. Mean of the RAVLT memory efficiency effect in the DMD was 1.8 in comparison to 2.0 of the control group, and was significantly reduced (p=0.008). Remaining variables were comparable to the age matched control group in this age group. However, in comparison to the respective control group, DMD '11-16' years group showed reduced immediate recall (p=0.024). Performance of '>16' years age group was comparable to the age matched control group. Performance of DMD '11-16' years and '>16' years age groups in the remaining RAVLT variables were comparable to their respective age matched control groups. Digit span backward and forward showed significant changes only in DMD '6-10' years age groups but comparable to that of respective control groups in the remaining age groups.

References

1. Prasad D, Verma S. PGI battery of brain dysfunctions. Agra, India: National Psychological Corporation. 1985.
2. Kar BR, Rao S, Chandramouli B, Thennarasu K. NIMHANS neuropsychological battery for children-manual. Bangalore: NIMHANS publication division. 2004.
3. Powell JB, Cripe LI, Dodrill CB. Assessment of brain impairment with the Rey Auditory Verbal Learning Test: A comparison with other neuropsychological measures. Archives of Clinical Neuropsychology. 1991;6(4):241-9.
4. Vakil E, Greenstein Y, Blachstein H. Normative data for composite scores for children and adults derived from the Rey Auditory Verbal Learning Test. Clin Neuropsychol. 2010;24(4):662-77.
5. Boone KB, Lu P, Wen J. Comparison of various RAVLT scores in the detection of noncredible memory performance. Archives of Clinical Neuropsychology. 2005;20(3):301-19.
6. Ricci M, Graef S, Blundo C, Miller LA. Using the Rey Auditory Verbal Learning Test (RAVLT) to differentiate alzheimer's dementia and behavioural variant fronto-temporal dementia. Clin Neuropsychol. 2012;26(6):926-41.
7. Golden CJ. The measurement of creativity by the Stroop Color and Word Test. Journal of personality assessment. 1975;39(5):502-6.
8. Golden CJ, Freshwater SM. Stroop color and word test. age. 1978;15:90.
9. Jensen AR, Rohwer WD. The Stroop color-word test: a review. Acta psychologica. 1966;25:36-93.
